# *Rumex japonicus* Houtt. Protects Dopaminergic Neurons by Regulating Mitochondrial Function and Gut–Brain Axis in In Vitro and In Vivo Models of Parkinson’s Disease

**DOI:** 10.3390/antiox11010141

**Published:** 2022-01-10

**Authors:** Hee-Young Kim, Chang-Hwan Bae, Jayoung Kim, Yukyoung Lee, Hyongjun Jeon, Hyungwoo Kim, Seungtae Kim

**Affiliations:** 1 Korean Medicine Research Center for Healthy Aging, Pusan National University, Yangsan 50612, Korea; kimhy@pusan.ac.kr; 2 Department of Korean Medical Science, School of Korean Medicine, Pusan National University, Yangsan 50612, Korea; ssaybch@gmail.com (C.-H.B.); dalkis0815@gmail.com (Y.L.); newace16@naver.com (H.J.); 3 Department of Convergence Medicine, School of Medicine, Pusan National University, Yangsan 50612, Korea; jyside@pusan.ac.kr; 4 Division of Pharmacology, School of Korean Medicine, Pusan National University, Yangsan 50612, Korea; kronos7@pusan.ac.kr

**Keywords:** *Rumex japonicus* Houtt., Parkinson’s disease, neurodegeneration, neuroinflammation, tight junction, α-synuclein, MPTP, MPP^+^, SH-SY5Y, gut–brain axis

## Abstract

Parkinson’s disease (PD) is the second most common neurodegenerative disease worldwide. *Rumex japonicus* Houtt. (RJ) has been used to treat gastrointestinal and inflammatory diseases in East Asia. However, it is unknown whether RJ can prevent PD. We investigated the neuroprotective effects of RJ in cellular and animal PD models, focused on mitochondrial function and the gut–brain axis. SH-SY5Y cells were treated with RJ (0.01 mg/mL) for 24 h, after which they were treated with the 1-methyl-4-phenylpyridinium ion (MPP^+^). MPP^+^-induced apoptosis increased mitochondrial reactive oxygen species and decreased ATP, PINK1, and DJ-1, which were inhibited by RJ. Ten-week-old C57BL/6N male mice were treated with 30 mg/kg of 1-methyl-4-phenyl-1,2,3,6-tetrahydropyridine (MPTP) for 5 days and orally administered 50 or 100 mg/kg of RJ for 14 days. RJ alleviated MPTP-induced behavioral impairment, dopaminergic neuronal death, and mitochondrial dysfunction in the substantia nigra (SN) and suppressed the MPTP-induced increase in lipopolysaccharide, interleukin-1β, tumor necrosis factor-α, α-synuclein, and apoptotic factors in the SN and colon. Moreover, RJ inhibited the MPTP-mediated disruption of the tight junction barrier in the colon and blood–brain barrier of mice. Therefore, RJ alleviates MPTP-induced inflammation and dopaminergic neuronal death by maintaining mitochondrial function and tight junctions in the brain and colon.

## 1. Introduction

Parkinson’s disease (PD) is an incurable neurodegenerative disease characterized by bradykinesia, tremor, muscle rigidity, and postural instability [1]. These PD symptoms are associated with the presence of Lewy bodies (composed of aggregates of α-synuclein fibrils) in the neuronal cytoplasm and progressive degeneration of dopaminergic (DA) neurons in the substantia nigra (SN) pars compacta [1]. The exact etiology of PD has not been determined; however, oxidative stress, inflammation, and mitochondrial dysfunction may be strongly associated with the progression of PD [2].

Recently, it has been reported that most PD patients have gastrointestinal (GI) disorders or symptoms, and GI disorders are one of the significant debilitating non-motor features of PD patients [3]. In addition, Lewy bodies or neurites in the enteric nervous system [4,5] and structural alterations of the intestinal epithelial barrier [6] have been found in PD patients. According to recent studies, the aggregation of α-synuclein seems to start in the gut; thus, it may be associated with abnormal gut conditions such as dysbiosis and gut barrier alteration in PD patients [7]. Therefore, the gut–brain axis is thought to be an important route in PD pathogenesis.

*Rumex japonicus* Houtt. (RJ), a perennial herb, is widely distributed in East Asia. The dried root of RJ has traditionally been used to treat many medical conditions, such as dermatopathy, jaundice, hematemesis, uterine bleeding, and gastrointestinal diseases [8,9]. RJ has several pharmaceutical effects, including anti-oxidative, anti-cancer, anti-inflammatory, anti-microbial, anti-diabetic, and anti-purgative effects [8,10]. Recently, we reported that the oral administration of RJ alleviated colonic inflammation by controlling tight junctions (TJs) and apoptosis in dextran sulfate sodium (DSS)-induced mice [11]. However, no studies have been conducted on the effects of RJ on PD. Therefore, we investigated the preventive or curative effects of RJ in PD using in vitro and in vivo studies that focused on its controlling effect on mitochondrial function and the gut–brain axis.

## 2. Materials and Methods

### 2.1. Sample Preparation

Dried RJ roots were purchased from GwangMeongDang Medicinal Herbs (Ulsan, Korea). The herb was harvested in Gyeongsanbukdo, South Korea, in 2015. All procedures of RJ methanol extraction were performed following our previous study [11]. The concentrated extract was lyophilized using a freeze dryer (Labconco, Kansas City, MO, USA), which produced 1.27 g of lyophilized powder (yield, 12.74%). A sample of the specimen was deposited in the herbarium at the Division of Pharmacology, School of Korean Medicine, Pusan National University (Voucher No. MH2012-006). Freeze-dried RJ was dissolved in water for oral administration to the mice.

### 2.2. Ultra-Performance Liquid Chromatography Coupled with Quadrupole-Time-of-Flight Tandem Mass Spectrometry (UPLC-Q-TOF-MS) Analysis

UPLC-Q-TOF-MS analysis using an Agilent 1290 Infinity LC system coupled with an Agilent 6530 Accurate-Mass-Q-TOF-LC/MS (Agilent Technologies, Santa Clara, CA, USA) was performed to confirm anthraquinones contained in the RJ extract. Chromatographic separations were performed using an ACQUITY BEH C18 column (2.1 mm × 100 mm, 1.7 μm, Waters, Milford, UK) maintained at 40 °C. The mobile phase consisted of 0.1% (*v*/*v*) formic acid and acetonitrile (solvent B) with a gradient program: 0–1.5 min, 0–10% B; 1.5–6 min, 10–17% B; 6–12 min, 17–43% B; 12–21 min, 43–95%; 21–21.1 min, 95–5% B; 21.1–25 min, 5% B; and maintained at 5% until stop. The flow rate was 0.3 mL/min, and the injection volume was 10 μL. The mass spectra were acquired in negative mode, *m*/*z* ranging from 100 to 1300, with electrospray ionization (ESI). The optimized MS conditions were as follows: gas flow 9 L/min, gas temperature 300 °C, nebulizer 45 psi, capillary voltage 4000 V, skimmer voltage 65 V, octopole RF voltage 750 V, fragmentor voltage 175 V. The obtained mass spectra were processed using Acquisition Software Version, 6200 series TOF/6500 series Q-TOF B.05.01 (B5125.1). The components were identified based on chromatographic retention time, formulation composition, references [12,13,14], and database PubChem (available online: https://pubchem.ncbi.nlm.nih.gov/ (accessed on 26 October 2021)).

### 2.3. Cell Culture

Human neuroblastoma SH-SY5Y cells (Korean Cell Line Bank, Seoul, Korea) were cultured in high-glucose Dulbecco’s modified Eagle’s medium (Welgene, Daegu, Korea) supplemented with 10% (*v*/*v*) heat-inactivated fetal bovine serum (Welgene) and 100 units/mL penicillin/streptomycin (Welgene). Cells were maintained at 37 °C in a humidified atmosphere of 5% CO_2_.

### 2.4. Cell Viability Assay

Cells were pre-incubated in 96-well plates at a confluence of 1 × 10^4^ cells/well and treated with RJ for 24 h, after which they were treated with the 1-methyl-4-phenylpyridinium ion (MPP^+^) for 24 h. The suitable dose of MPP^+^ (1 mM) for further experiments was determined by pilot tests and previous studies [15,16]. They were then treated with 0.5 mg/mL of 3-(4,5-dimethyl-thiazol-2-yl)-2,5-diphenyl tetrazolium bromide (MTT; Duchefa Biochemie, Haarlem, The Netherlands) for 4 h. After the medium was discarded, 100 μL of dimethyl sulfoxide was added to dissolve the purple formazan crystals. Following incubation for 30 min, the plates were read at a 540 nm wavelength.

### 2.5. ATP Assay

Cells were cultured in 6-well plates at a confluence of 1 × 10^6^ cells/well with or without RJ for 24 h and then incubated with or without MPP^+^ for 24 h. Collected cells were lysed, and the cell protein was precipitated and neutralized using a Deproteinizing Sample Preparation Kit (Biovision, Milpitas, CA, USA). Then, a 96-well plate containing 50 μL of sample and 50 μL of the reaction mixture of the ATP assay kit (Biovision) was incubated at RT for 30 min and read at 570 nm. The amount of ATP in the sample was calculated using a standard curve.

### 2.6. Measurement of Mitochondrial Reactive Oxygen Species (ROS)

Cells were cultured in 8-well chamber slides with or without RJ for 24 h, then re-incubated with or without MPP^+^ for 24 h, after which 5 μM MitoSOX red mitochondrial superoxide indicator (Invitrogen, Carlsbad, CA, USA) was added, and the samples were incubated for 10 min at 37 °C. The cells were washed and fixed with 4% (*v*/*v*) paraformaldehyde (PFA, 25 min, 4 °C) and mounted with Vectashield mounting medium with 4′,6-diamidino-2-phenylindole (DAPI) (Vector, Burlingame, CA, USA). Images of the slides were captured using a Zeiss Axio Imager M1 microscope (Zeiss, Oberkochen, Germany).

### 2.7. Flow Cytometric Analysis

Cells were cultured in 6-well plates at a confluence of 4 × 10^5^ cells/well with or without RJ for 24 h and incubated with or without MPP^+^ for 24 h. Cells were collected by centrifugation and washed with phosphate-buffered saline (PBS). Fluorescein isothiocyanate-labeled annexin V and propidium iodide (BD Bioscience, San Jose, CA, USA) were added to the cells, which were re-suspended in annexin V binding buffer. After incubation (15 min, RT), the cells were analyzed using a BD FACSCanto II Flow Cytometer (BD Biosciences).

### 2.8. Animal Study Design

C57BL/6N mice (male, 10 weeks old) were obtained from Samtaco Bio Korea (Osan, Korea). The mice were maintained under controlled conditions (relative humidity 55 ± 5%, 22 ± 2 °C, 12 h light/dark cycle), and feed and drinking water were supplied ad libitum.

The mice were adapted for 1 week and then randomly divided into four groups: normal, MPTP, RJ-L, and RJ-H. Mice in the MPTP, RJ-L, and RJ-H groups were administered MPTP-HCl (30 mg/kg, Sigma, St. Louis, MO, USA) dissolved in saline intraperitoneally for the first 5 days. Mice in the normal and MPTP groups were orally administered the vehicle (water), and the mice in the RJ-L and RJ-H groups were administered 50 mg/kg (RJ-L) or 100 mg/kg (RJ-H) doses of RJ for 14 days. The proper administration doses of RJ were determined by a pilot test and a previous study [11]. Behavior tests were conducted on days 0, 5, and 13. All mice were euthanized with isoflurane and sacrificed on day 14. The study protocol was approved by the Institutional Animal Care and Use Committee of Pusan National University (Busan, Korea; approval number PNU-2018-1847).

### 2.9. Behavior Test

To confirm the behavioral changes, pole and rotarod tests were conducted. All mice were trained for three days before starting the experiment. The behavior tests were repeated three times, and the mean value of the three sessions was used.

For the pole test, mice were placed head-downwards near the top of a gauze-wrapped acrylic pole (10 mm in diameter and 55 cm in height), and the time taken to reach the bottom of the pole was measured.

For the rotarod test, the mice were placed on a bumpy surface rotating cylinder (Harvard Apparatus, Holliston, MA, USA), and the staying time was measured. The initial rotating speed of the cylinder was 4 rpm, which was increased to 40 rpm for 2 min, and the cut-off time was 3 min.

### 2.10. Immunohistochemistry

The SN tissues from the mice were fixed with 4% PFA for 48 h, immersed in 30% (*w*/*v*) sucrose solution, and sectioned at a thickness of 30 μm. The free-floating sections were incubated with 1% (*v*/*v*) H_2_O_2_ in 0.5 M PBS for 15 min, blocked with 5% (*v*/*v*) normal goat serum (NGS) for 1 h, and incubated with anti-tyrosine hydroxylase (TH) primary antibody (Santa Cruz Biotechnology, Santa Cruz, CA, USA) overnight at 4 °C. The sections were incubated with Vectastain Elite ABC reagents (Vector) for 1.5 h at RT, incubated with Diaminobenzidine Substrate Kit (Vector) for 10 min at RT, and then mounted on gelatin-coated slides. Images of TH-positive cells in the SN were captured using an Axio Scope A1 microscope and AxioCam ICc3 camera (Zeiss). The TH-positive cells were counted on each capture and confirmed three times.

### 2.11. Western Blot

SH-SY5Y cells or tissues (colon and SN) were lysed with RIPA buffer (Invitrogen) and centrifuged at 13,000 rpm for 15 min at 4 °C. Total protein concentration was determined using a Bio-Rad protein assay kit (Bio-Rad Laboratories, Inc., Hercules, CA, USA). The proteins were separated by sodium dodecyl sulfate–polyacrylamide gel electrophoresis and electro-transferred to a 0.45 μm nitrocellulose blotting membrane (GE Healthcare UK Ltd., Little Chalfont, UK). The blots were incubated overnight at 4 °C with a primary antibody immersed in 5% (*w*/*v*) bovine serum albumin (BSA) or 5% (*w*/*v*) skim milk. The blots were then incubated with a secondary antibody for 1 h at RT. The membranes were then washed three times with PBS containing 0.05% (*v*/*v*) Tween 20 and visualized with an enhanced chemiluminescence reagent (Thermo Fisher Scientific Inc., Rochford, IL, USA). The primary antibodies used for Western blotting were anti-Bcl-2, anti-cytochrome c, anti-caspase-3, anti-TH, anti-PTEN-induced putative kinase 1 (PINK1), anti-Parkin, anti-DJ-1, anti-zona occludens (ZO)-1, anti-occludin, anti-vascular endothelial (VE)-cadherin, anti-β-actin (all from Santa Cruz Biotechnology), anti-Bax (Abcam, Cambridge, UK), anti-cleaved caspase-3 (Cell Signaling Technology Inc., Beverly, MA, USA), and anti-claudin-5 (Invitrogen). The blots were quantified using ImageJ software (National Institutes of Health, Bethesda, MD, USA, available online: www.imagej.nih.gov (accessed on 8 March 2021)).

### 2.12. Immunofluorescence

SH-SY5Y cells cultured in 8-well chamber slides were fixed with 4% PFA for 20 min and then permeabilized with 0.1% (*v*/*v*) Triton X-100 in PBS for 15 min at RT. The cells were then blocked with 10% NGS in PBS for 1 h at RT.

The colon tissues from the mice were fixed with 4% PFA for 48 h and immersed in 30% (*w*/*v*) sucrose solution. The tissues were embedded in cryomolds (Sungwon Medical Co., Seoul, Korea) with optimal cutting temperature compound (Sakura Finetek, Torrance, CA, USA) at −80 °C and sectioned at 20 μm thickness. The colon sections were attached to a glass slide and fixed in 4% PFA for 15 min. Then, the sections were blocked in blocking buffer (5% NGS and 0.3% (*v*/*v*) Triton X-100 containing PBS) for 1 h at RT.

The NGS-blocked slides (containing cells or tissue sections) were subsequently incubated with anti-PINK1, anti-Parkin, anti-DJ-1, anti-ZO-1, and anti-occludin primary antibodies (Santa Cruz Biotechnology) dissolved in 10% NGS or antibody dilution buffer (5% NGS, 1% BSA (*w*/*v*), and 0.3% Triton X-100 containing PBS) overnight at 4 °C. They were then incubated with anti-rabbit or anti-mouse Alexa-488 IgG (Molecular Probes, Eugene, OR, USA) or anti-mouse Alexa-594 IgG (Molecular Probes) fluorophore-conjugated secondary antibodies for 1 h at RT in the dark. The slides were rinsed with PBS and mounted with Vectashield mounting medium with DAPI (Vector), after which images of the slides were captured using a Zeiss Axio Imager M1 microscope (Zeiss). All signal-positive cells were counted for each capture and confirmed three times.

### 2.13. Enzyme-Linked Immunosorbent Assay (ELISA)

To prepare the samples, the colon and SN tissue pieces were homogenized in PBS and sonicated on ice. The supernatant was collected after centrifugation at 13,000 rpm for 5 min. Mouse blood was centrifuged (3000 rpm, 10 min), and serum was collected. The changes in lipopolysaccharide (LPS; Abbkine Inc., Wuhan, China), TNF-α (Biolegend, San Diego, CA, USA), IL-1β (Biolegend), and α-synuclein (LSbio, Seattle, WA, USA) were confirmed according to the manufacturer’s protocols.

### 2.14. Statistical Analysis

All data are indicated as the means ± standard deviation. Behavioral tests were assessed using paired *t*-tests and Duncan’s multiple range tests, and all data, except the behavioral tests, were analyzed using the one-way analysis of variance with Tukey’s honestly significant difference tests using SPSS statistics Ver. 25 (IBM, Armonk, NY, USA). Differences were considered statistically significant at *p* < 0.05.

## 3. Results

### 3.1. RJ Contains Anthraquinone Substrates

Anthraquinones are known to play an important role in RJ. Therefore, we confirmed that anthraquionone compounds were contained in the extract RJ used in the present study by UHPLC-QTOF-MS analysis. The RJ contained 8 major anthraquinone compounds: emodin (*m*/*z* 269.0456), aloe emodin (*m*/*z* 269.0458), emodin-8-glucoside (*m*/*z* 431.0987), chrysohanol (*m*/*z* 253.0505), chrysophanol-8-O-β-d-glucoside (*m*/*z* 415.1030), physcion (*m*/*z* 283.0612), physcion-8-O-β-d-glucopyranoside (*m*/*z* 445.1140), and rhein (*m*/*z* 283.0255) (Table 1).

### 3.2. RJ Protects SH-SY5Y Cells against MPP^+^-Induced Cytotoxicity and Mitochondrial Dysfunction

**Figure 1:**
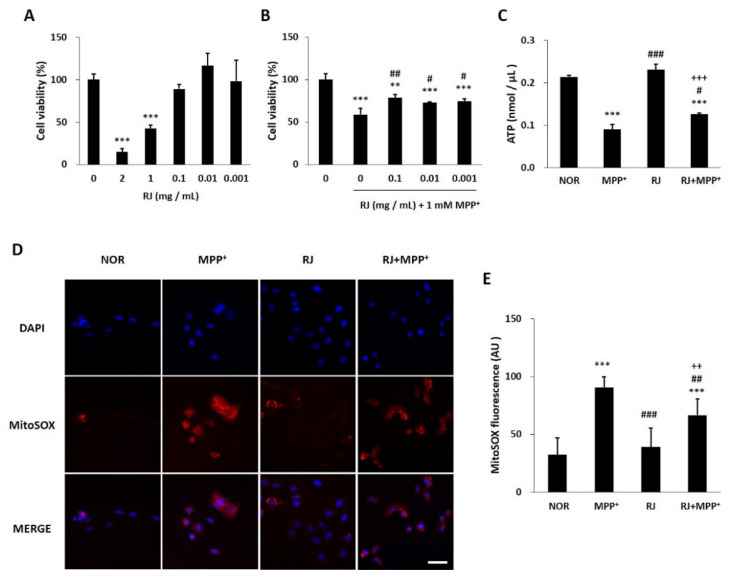
Cell viability and mitochondrial generation of ATP and reactive oxidative species (ROS) in *Rumex japonicus* Houtt. (RJ)-treated SH-SY5Y cells. (**A**,**B**) Cell viability of SH-SY5Y cells exposed to various doses of RJ for 24 h and 1 mM 1-methyl-4-phenylpyridinium ion (MPP^+^) for 24 h together with the RJ. (**C**) The levels of ATP. (**D**,**E**) The levels of mitochondrial ROS in MPP^+^-treated cells. The experiment was repeated 3 times and 5 figures per group were quantified and are presented as the mean ± standard deviation. Values are presented as the mean ± standard deviation. Mean values were significantly different (** *p* < 0.01 and *** *p* < 0.001, compared with the normal group; ^#^ *p* < 0.05, ^##^ *p* < 0.01 and ^###^ *p* < 0.001, compared with the group treated by MPP^+^ alone; ^++^ *p* < 0.01 and ^+++^ *p* < 0.001, compared with the group treated by RJ alone) as determined by Tukey’s honestly significant difference test. Scale bar = 50 μm.

As a result of evaluating the cytotoxicity of RJ, three doses (0.1, 0.01, and 0.001 mg/mL) were non-toxic to SH-SY5Y cells (Figure 1A). The doses also significantly attenuated MPP^+^ toxicity in the cells (Figure 1B). Based on the result of the two MTT assays, a dose of 0.01 mg/mL RJ was selected and used in further experiments.

MPP^+^ treatment significantly decreased the ATP level in the cells (*p* < 0.001); however, RJ pretreatment partially attenuated the MPP^+^-induced ATP reduction (*p* < 0.05, Figure 1C).

The MitoSOX red fluorescent signal appeared in the oxidized mitochondria of live cells. In this study, MPP^+^ significantly increased MitoSOX red signal in SH-SY5Y cells (*p* < 0.001), which was partially attenuated by RJ pretreatment (*p* < 0.01). In addition, RJ pretreatment did not induce changes in the red signal in normal cells. These results indicate that RJ pretreatment inhibits MPP^+^-induced ROS generation in the mitochondria of SH-SY5Y cells (Figure 1D,E).

### 3.3. RJ Inhibits MPP^+^-Mediated Reduction in Parkin, PINK1, and DJ-1 in SH-SY5Y Cells

**Figure 2:**
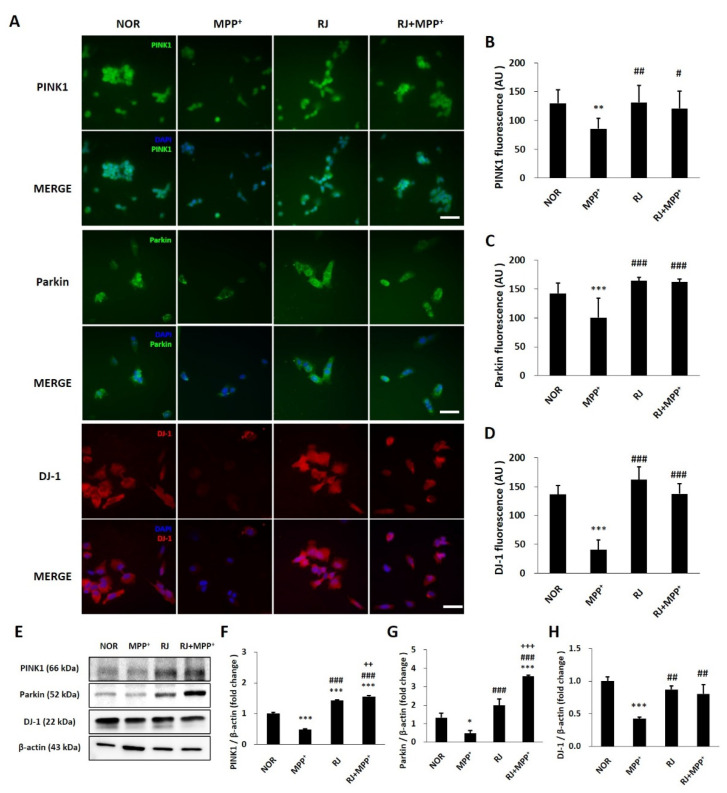
*Rumex japonicus* Houtt. (RJ) attenuated 1-methyl-4-phenylpyridinium ion (MPP^+^)-mediated reduction in Parkinson’s disease (PD)-related mitochondrial proteins in SH-SY5Y cells. (**A**–**D**) Cells treated with RJ with/without MPP^+^ were stained with antibodies of PINK1, Parkin, and DJ-1. The immunofluorescence experiment was repeated 3 times and 5 figures per group were quantified. (**E**–**H**) Changes in PINK1, Parkin and DJ-1 were confirmed by Western blotting (*n* = 3). Values are presented as the mean ± standard deviation. Mean values were significantly different (* *p* < 0.05, ** *p* < 0.01 and *** *p* < 0.001, compared with the normal group; ^#^ *p* < 0.05, ^##^ *p* < 0.01 and ^###^ *p* < 0.001, compared with the group treated by MPP^+^ alone; ^++^ *p* < 0.01 and ^+++^ *p* < 0.001, compared with the group treated by RJ alone) as determined by Tukey’s honestly significant difference test. Scale bar = 50 μm.

The proteins Parkin, PINK1, and DJ-1 are important factors in maintaining mitochondrial function. MPP^+^ reduced the fluorescent signals of Parkin (*p* < 0.01), PINK1 (*p* < 0.001), and DJ-1 (*p* < 0.001) in the cells; however, RJ pretreatment significantly inhibited the MPP^+^-induced reduction in PINK1 (*p* < 0.05), Parkin (*p* < 0.001), and DJ-1 (*p* < 0.001) in SH-SY5Y cells. RJ pretreatment did not influence the Parkin, PINK1, and DJ-1 signals in normal cells (Figure 2A–D). These results were confirmed by Western blot analysis (Figure 2E–H).

### 3.4. RJ Partially Protects SH-SY5Y Cells against MPP^+^-Induced Apoptosis

**Figure 3:**
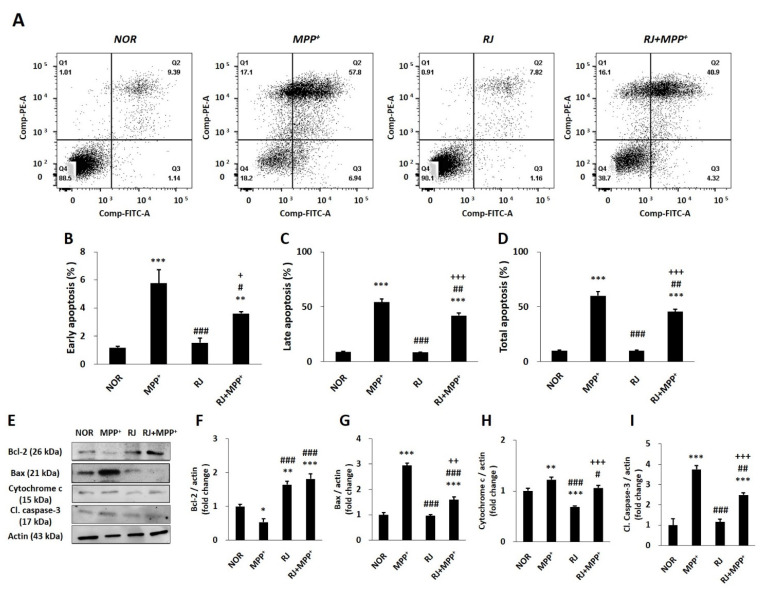
*Rumex japonicus* Houtt. (RJ) inhibited 1-methyl-4-phenylpyridinium ion (MPP^+^)-induced apoptosis in SH-SY5Y cells. (**A**–**D**) Apoptosis of the cells treated with RJ with/without MPP^+^ were confirmed by cytometry and quantified in the early, late, and total stages. (**E**–**I**) The expressions of Bcl-2, Bax, cytochrome c, and caspase-3 were confirmed by Western blotting. Values are expressed as the mean ± standard deviation (*n* = 3). Mean values were significantly different (* *p* < 0.05, ** *p* < 0.01 and *** *p* < 0.001, compared with the normal group; ^#^ *p* < 0.05, ^##^ *p* < 0.01 and ^###^ *p* < 0.001, compared with the group treated by MPP^+^ alone; ^+^ *p* < 0.05, ^++^ *p* < 0.05 and ^+++^ *p* < 0.001, compared with the group treated by RJ alone) as determined by Tukey’s honestly significant difference test.

Flow cytometry analysis showed that MPP^+^ treatment increased early (*p* < 0.001) and late (*p* < 0.001) apoptosis; however, pretreatment with RJ effectively inhibited both early (*p* < 0.05) and late (*p* < 0.01) apoptosis in MPP^+^-treated cells (Figure 3A–D).

The expression of Bcl-2 was decreased in response to MPP^+^ treatment (*p* < 0.05); however, RJ increased Bcl-2 expression in both the normal (*p* < 0.01 in each group) and MPP^+^-treated cells (*p* < 0.001 for each group, Figure 3E,F). MPP^+^ significantly increased Bax (*p* < 0.001), caspase-3 (*p* < 0.01), and cytochrome c (*p* < 0.001) in the cells; however, RJ pretreatment significantly inhibited the MPP^+^-induced increase in Bax (*p* < 0.01 in each group), caspase-3 (*p* < 0.01 at each group), and cytochrome c (*p* < 0.05; Figure 3E,G–I).

### 3.5. RJ Improves the Abnormal Behavior of MPTP-Treated Mice

**Figure 4:**
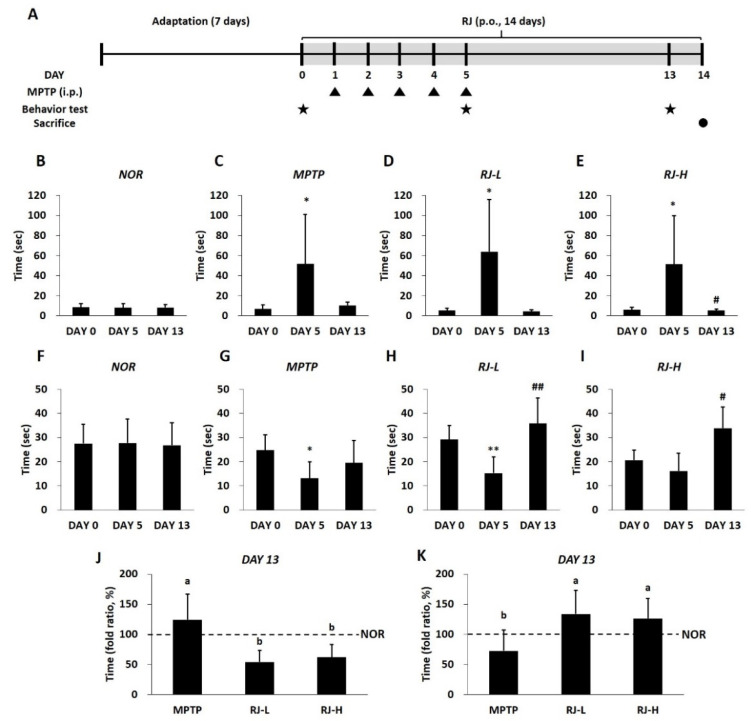
*Rumex japonicus* Houtt. (RJ) alleviated 1-methyl-4-phenyl-1,2,3,6-tetrahydropyridine (MPTP)-induced motor dysfunction in mice with Parkinson’s disease. (**A**) In vivo study design of Parkinson’s disease. The behavior changes were confirmed by the pole test (**B**–**E**) and the rotarod test (**F**–**I**). Comparison between the groups on day 13 in the pole test (**J**) and the rotarod test (**K**). Values are expressed as the mean ± standard error of mean (*n* = 6). Mean values were significantly different as determined by the paired *t*-test (* *p* < 0.05 and ** *p* < 0.01, between day 0 and 5; ^#^ *p* < 0.05, ^##^ *p* < 0.01, between day 5 and 13) or Duncan’s multiple range tests (different lowercase letters (a, b) were significantly different (*p* < 0.05)).

The in vivo study design of the present study is shown in Figure 4A. To confirm the behavioral changes, pole and rotarod tests were conducted. In the pole test, there was no significant difference in the normal group in the time taken to arrive at the bottom of the pole on experimental days; however, the time was significantly increased in the MPTP, RJ-L, and RJ-H groups (*p* < 0.05) on day 5 (the final day of MPTP injection). On day 13, the time in the RJ-H group was significantly reduced compared with the time in each group on day 5 (*p* < 0.05, Figure 4B–E). In the rotarod test, the staying time on the cylinder was not significantly different in the normal group during the experimental period. The duration of stay in the MPTP and RJ-L groups on day 5 was significantly shorter than that on day 0 (*p* < 0.05 and *p* < 0.01, respectively). The time in the MPTP group on day 13 was not significantly different from that on day 5; however, the times in the RJ-L (*p* < 0.01) and RJ-H (*p* < 0.05) groups were significantly longer than those on day 5 (Figure 4F–I). On day 13, RJ-L and RJ-H groups showed a shorter time to descend in the pole test (Figure 4J), and a longer time to stay in the cylinder in the rotarod test (Figure 4K) than the MPTP group (*p* < 0.05 in each group).

### 3.6. RJ Inhibits the Loss of TH and Mitochondrial Factors of MPTP-Treated Mice

**Figure 5:**
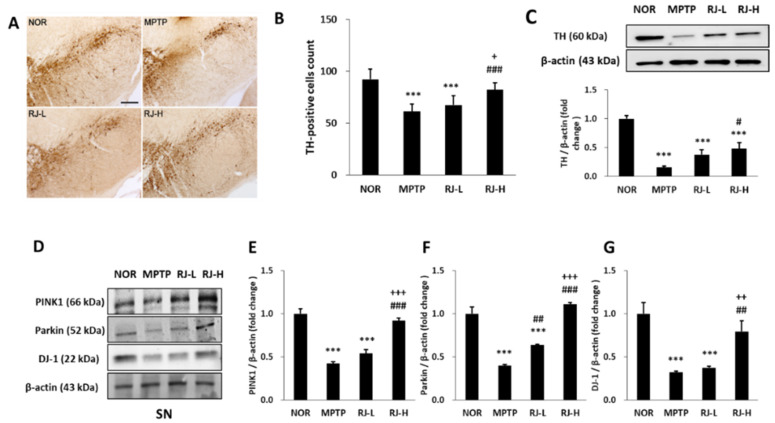
*Rumex japonicus* Houtt. (RJ) attenuated the loss of tyrosine hydroxylase (TH)-positive cells in the substantia nigra (SN) and Parkinson’s disease (PD)-related mitochondrial proteins in the SN of PD mice. (**A**,**B**) Changes in 1-methyl-4-phenyl-1,2,3,6-tetrahydropyridine (MPTP)-induced loss of tyrosine hydroxylase (TH)-positive cells in immunohistochemical observation. (**C**) The protein expressions of TH in the SN. (**D**–**G**) The mitochondrial factors in SN including PINK1, Parkin and DJ-1. Values are expressed as the mean ± standard deviation (*n* = 3). Mean values were significantly different (*** *p* < 0.001, compared with the normal group; ^#^ *p* < 0.05, ^##^ *p* < 0.01 and ^###^ *p* < 0.001, compared with the MPTP group; ^+^ *p* < 0.05, ^++^ *p* < 0.01 and ^+++^ *p* < 0.001, compared with the RJ-L group) by Tukey’s honestly significant difference test. Scale bar = 200 μm.

The number of TH-positive cells in the MPTP and RJ-L groups was significantly reduced compared with that in the normal group (*p* < 0.001 in each group); however, the number of TH-positive cells in the RJ-H group was significantly higher than those in the MPTP (*p* < 0.001) and RJ-L (*p* < 0.05) groups (Figure 5A,B), and the result of Western blotting also showed that the TH expression in the RJ-H group was significantly higher than that in the MPTP group (*p* < 0.05; Figure 5C). Moreover, MPTP suppressed PINK1, Parkin, and DJ-1 expression in the SN (*p* < 0.001 for each protein), and RJ prevented them (*p* < 0.001, *p* < 0.01, and *p* < 0.01, respectively; Figure 5D–G). This tendency is similar to the results of the in vitro experiments (Figure 2) conducted prior to the animal study.

### 3.7. RJ Regulates Apoptosis in the Colon and the SN of MPTP-Treated Mice

**Figure 6:**
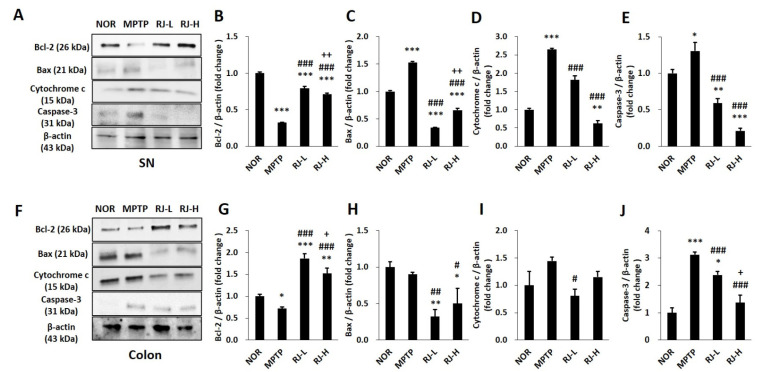
*Rumex japonicus* Houtt. (RJ) inhibited 1-methyl-4-phenyl-1,2,3,6-tetrahydropyridine (MPTP)-induced apoptosis in the substantia nigra (SN) and the colon of mice. (**A**–**E**) The protein expressions of apoptotic factors in the SN, including Bcl-2, Bax, cytochrome c, and cleaved caspase-3. (**F**–**J**) The same apoptotic factors of the SN and colon. Values are expressed as the mean ± standard deviation (*n* = 3). Mean values were significantly different (* *p* < 0.05, ** *p* < 0.01 and *** *p* < 0.001, compared with the normal group; ^#^ *p* < 0.05, ^##^ *p* < 0.01 and ^###^ *p* < 0.001, compared with the MPTP group; ^+^ *p* < 0.05 and ^++^ *p* < 0.01, compared with the RJ-L group) as determined by Tukey’s honestly significant difference test.

MPTP-induced mitochondrial dysfunction affects mitochondria-dependent apoptosis. In the SN, the MPTP group showed a decrease in Bcl-2 and an increase in Bax, cytochrome c, and cleaved caspase-3; however, the RJ-L and RJ-H groups showed higher levels of anti-apoptotic Bcl-2 (*p* < 0.001 in each group) and lower levels of pro-apoptotic Bax, cytochrome c, and cleaved caspase-3 (*p* < 0.001 in each group, Figure 6A–E) than the MPTP group. Interestingly, a similar tendency of apoptotic protein expression was observed in the colon. The MPTP group showed a significant decrease in Bcl-2 (*p* < 0.05) and an increase in cleaved caspase-3 (*p* < 0.001) compared with the normal group; however, groups with low or high doses of RJ showed significantly higher levels of Bcl-2 (*p* < 0.001 in each group) and lower levels of cleaved caspase-3 (*p* < 0.001 in each group) than the MPTP group. MPTP also increased the level of cytochrome c, which was prevented by low doses of RJ (*p* < 0.05, Figure 6F–J).

### 3.8. RJ Suppresses the Loss of TJs in the Colon and the SN of MPTP-Treated Mice

**Figure 7:**
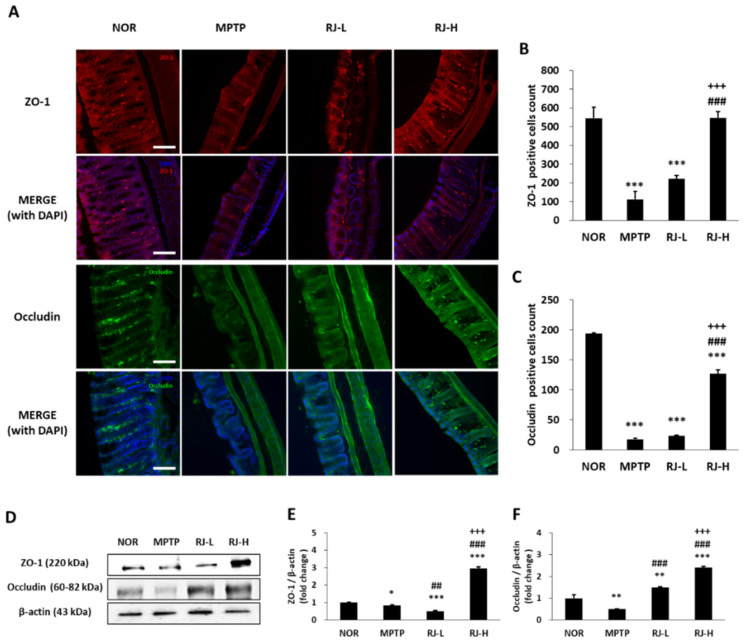
*Rumex japonicus* Houtt. (RJ) suppressed collapse of tight junction (TJ) barrier in the colon of a mouse model of Parkinson’s disease. (**A**–**C**) Changes in colonic TJs including ZO-1 and occludin in immunofluorescence assays. (**D**–**F**) The protein expressions of ZO-1 and occludin. Values are expressed as mean ± standard deviation (*n* = 3). Mean values were significantly different (* *p* < 0.05, ** *p* < 0.01 and *** *p* < 0.001, compared with the normal group; ^##^ *p* < 0.01 and ^###^ *p* < 0.001, compared with the MPTP group; ^+++^ *p* < 0.001, compared with the RJ-L group) as determined by Tukey’s honestly significant difference test. Scale bar = 50 μm.

The colon tissues of the normal group showed normal mucosal structure, fine crypts, and abundant ZO-1- and occludin-positive cells. However, the colon of the MPTP group showed a collapse of the colonic structure, the loss of TJs, and reduction in ZO-1- and occludin-positive cells. High doses of RJ suppressed the MPTP-induced abnormal structural changes (Figure 7A) and the loss of ZO-1- (*p* < 0.001) and occludin-positive cells (*p* < 0.001, Figure 7A–C).

The expression of proteins associated with colonic TJs was confirmed using Western blotting. The MPTP and RJ-L groups showed lower levels of ZO-1 than the normal group, but the RJ-H group showed higher expression than the other groups (*p* < 0.001, Figure 7D,E). The level of occludin in the MPTP group was lower than that in the normal group; however, the levels of occludin in the RJ-L and RJ-H groups were higher than those in the normal and MPTP groups (Figure 7D,F). These results indicate that RJ effectively suppressed the MPTP-induced loss of TJs.

### 3.9. RJ Reduces the Levels of LPS and Pro-Inflammatory Cytokines in MPTP-Treated Mice

**Figure 8:**
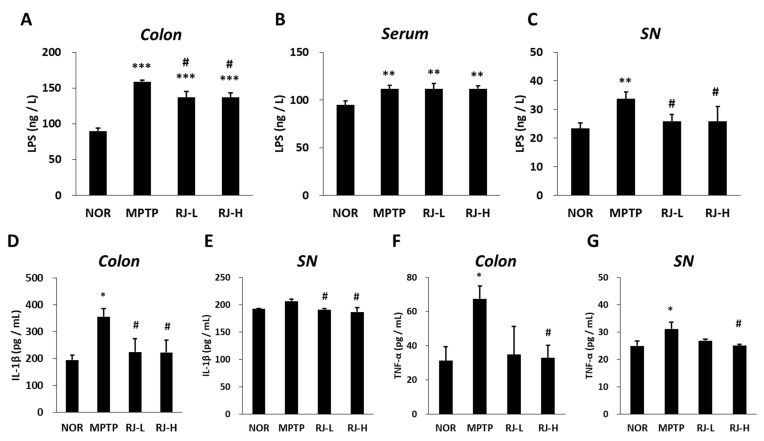
*Rumex japonicus* Houtt. (RJ) decreased the levels of lipopolysaccharide (LPS) and pro-inflammatory cytokines in the colon, serum, or substantia nigra (SN) of a mouse model of Parkinson’s disease. (**A**–**C**) The changes in the level of LPS in the colon, serum, and SN. (**D**,**E**) Interleukin (IL)-1β in the colon and the SN. (**F**,**G**) Tumor necrosis factor (TNF)-α in the colon and the SN. Values are expressed as mean ± standard deviation (*n* = 3). Mean values were significantly different (* *p* < 0.05 and ** *p* < 0.01 and *** *p* < 0.001, compared with the normal group; ^#^ *p* < 0.05, compared with the MPTP group) as determined by Tukey’s honestly significant difference test.

The concentration of colonic LPS was significantly higher in the MPTP-treated group (*p* < 0.001 for each group) than in the normal group; however, the RJ-L and RJ-H groups showed significantly lower LPS levels than the MPTP group (*p* < 0.05, Figure 8A). In the serum, MPTP-treated groups showed higher LPS levels than the normal group (*p* < 0.01 in each group; Figure 8B). In the SN, the MPTP group showed the highest level of LPS; however, the RJ-treated group showed significantly lower LPS levels than the MPTP group (*p* < 0.05 for each group, Figure 8C).

The level of IL-1β in the MPTP group was higher in the colon than in the normal group; however, there was no significant change in the SN. Low and high doses of RJ effectively inhibited the MPTP-mediated increase in IL-1β (*p* < 0.05 in each group, Figure 8D,E). In addition, the MPTP group showed significantly higher levels of TNF-α than the normal group in both the colon and SN tissues (*p* < 0.05 at each tissue); however, the RJ-H group showed significantly lower levels than the MPTP group in the tissues (*p* < 0.05, Figure 8F,G).

### 3.10. RJ Suppresses Increase in α-Synuclein in MPTP-Treated Mice

**Figure 9:**
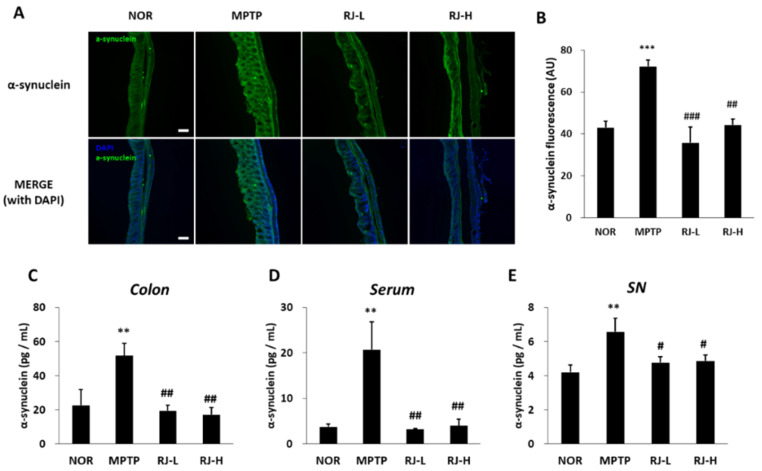
*Rumex japonicus* Houtt. (RJ) attenuated the increase in α-synuclein levels in the colon, serum, and substantia nigra (SN) of a mouse model of Parkinson’s disease. (**A**,**B**) The changes in the level of colonic α-synuclein by immunofluorescence assay. (**C**–**E**) The levels of α-synuclein in the colon, serum, and SN by enzyme-linked immunosorbent assay. Values are expressed as mean ± standard deviation (*n* = 3). Mean values were significantly different (** *p* < 0.01 and *** *p* < 0.001, compared with the normal group; ^#^ *p* < 0.05, ^##^ *p* < 0.01 and ^###^ *p* < 0.001, compared with the MPTP group) as determined by Tukey’s honestly significant difference test. Scale bar = 100 μm.

α-synuclein is a major factor in PD pathology. Therefore, changes in the level of α-synuclein were confirmed by immunofluorescence and ELISA. In immunofluorescent observation of the colon tissue, the MPTP group showed significantly higher levels of α-synuclein than the normal group; however, the RJ-L (*p* < 0.001) and RJ-H (*p* < 0.01) groups showed significantly lower levels than the MPTP group (Figure 9A,B). In the ELISA assay, the MPTP group showed the highest concentration of α-synuclein in the colon, serum, and SN (*p* < 0.01, Figure 9C–E); however, the RJ-treated groups showed significantly lower levels of α-synuclein than the MPTP group in the colon (*p* < 0.01), serum (*p* < 0.01), and SN (*p* < 0.05, Figure 9C–E).

### 3.11. RJ Prevents MPTP-Induced Disruption of the Blood–Brain Barrier (BBB) TJs

**Figure 10:**
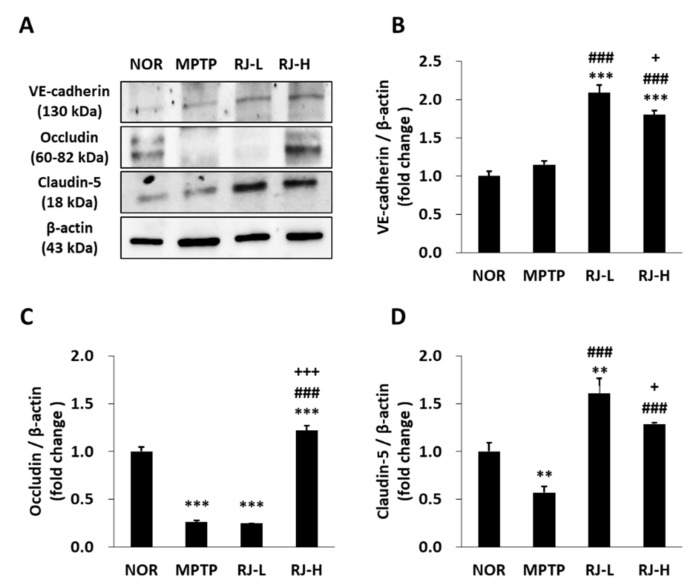
*Rumex japonicus* Houtt. (RJ) inhibited the 1-methyl-4-phenyl-1,2,3,6-tetrahydropyridine (MPTP)-mediated weakness of the blood–brain barrier (BBB) in a mouse model of Parkinson’s disease (PD). (**A**–**D**) The protein expressions of the BBB-related proteins, including VE-cadherin, occludin, and claudin-5 in the SN of PD mice. Values are expressed as mean ± standard deviation (*n* = 3). Mean values were significantly different (** *p* < 0.01 and *** *p* < 0.001, compared with the normal group; ^###^ *p* < 0.001, compared with the MPTP group; ^+^ *p* < 0.05 and ^+++^ *p* < 0.001, compared with the RJ-L group) as determined by Tukey’s honestly significant difference test.

The expression of adherens junctions (AJs) and TJ-related factors in the SN was confirmed by Western blotting. Mice in the RJ-L and RJ-H groups showed a significantly higher expression of vascular endothelial (VE)-cadherin, a member of the AJs, than the normal and MPTP groups (*p* < 0.001, in each group; Figure 10A,B). Moreover, MPTP reduced the expression of occludin (*p* < 0.001) and claudin-5 (*p* < 0.01), and a low dose of RJ only inhibited the reduction in claudin-5 (*p* < 0.001); however, a high dose of RJ inhibited the reduction in occludin (*p* < 0.01) and claudin-5 (*p* < 0.001, Figure 10A,C,D).

## 4. Discussion

In the present study, we demonstrated the neuroprotective effect of RJ, a medicinal herb commonly used in GI disease, in PD models focused on mitochondrial function and the gut–brain axis.

RJ contains various functional substances: anthraquinones, phytosterols, phenolic compounds, flavonoids, and so on [14]. Anthraquinones are known to play an important role in RJ [14]. Emodin, aloe–emodin, chrysophanol, rhein, physcion, and their derivatives are major components in anthraquinones, which play significant anti-cancer, anti-inflammation, anti-bacteria, and liver-protective roles [13,14]. The methanolic extract of RJ used in the present study also contained eight kinds of major anthraquinones (Table 1).

The neurotoxin MPP^+^, a metabolite of MPTP, induces oxidative stress [17], mitochondrial dysfunction [18], and the degeneration of DA neurons. PINK1, Parkin, and DJ-1 are involved in cell apoptosis and various mitochondrial functions, such as ATP production, mitochondrial respiration, changes in mitochondrial morphology, mitochondrial membrane potential, mitophagy, and mitochondrial transportation [2]. PINK1 recruits Parkin to the outer membrane of damaged mitochondria to repair the damage [2], and Parkin suppresses DA neuronal death by suppressing α-synuclein aggregation [19]. DJ-1 also prevents mitochondrial fragmentation and protects neuronal cells from toxins [2]. In the present study, pretreatment with RJ significantly alleviated MPP^+^-mediated reduction in ATP production (*p* < 0.05, Figure 1C) and the accumulation of ROS in the mitochondria of SH-SY5Y cells (*p* < 0.01, Figure 1D,E). Moreover, RJ effectively prevented the reduction in PINK1, Parkin, and DJ-1 in MPP^+^-treated SH-SY5Y cells (Figure 2), as well as in MPTP-treated mice (Figure 5D–G), indicating that RJ can alleviate MPP^+^-induced mitochondrial dysfunction in DA neurons.

The Bcl-2 family is closely associated with oxidative-stress-mediated apoptosis by regulating mitochondrial membrane permeability and the release of cytochrome c from the mitochondria to the cytosol [20]. Once oxidative stress attacks the cells, anti-apoptotic Bcl-2 reduces, whereas pro-apoptotic Bax increases, which induces the release of cytochrome c. The released cytochrome c activates caspase-3, and finally, the cells undergo apoptosis [21]. In the present study, treatment with MPP^+^-induced apoptosis in SH-SY5Y cells; however, treatment with RJ alleviated MPP^+^-induced apoptosis by controlling the expression of Bcl-2, Bax, cytochrome c, and caspase-3 (Figure 3). Taken together, oral treatment with RJ effectively inhibited mitochondria-dependent apoptosis in the SN and colon of MPTP-treated mice (Figure 6), indicating that RJ inhibits apoptosis by regulating the Bcl-2 family.

Abnormal colonic conditions such as inflammation, abnormal bacterial composition, increased α-synuclein, and increased intestinal permeability (the so-called leaky gut) were found in PD patients [22]. In particular, increased intestinal permeability is detected in PD patients and even in patients with no GI dysfunction [22,23]. The intestinal epithelial barrier maintains homeostasis by preventing the passage of toxic compounds while allowing the absorption of nutrients to the intestine [6]. The intestinal epithelial barrier is composed of TJs, AJs, gap junctions, and desmosomes, and TJs are mainly involved in the regulation of inter- and paracellular transport [24]. TJs are composed of transmembrane proteins, such as claudins and occludin, and framework proteins, such as ZO proteins [6]. Claudins and occludin are the most important regulators of cell barrier function, and ZO proteins form the central network for protein interactions [24]. In particular, occludin controls paracellular permeability by binding to ZO-1, which mediates the interaction between proteins and connects to the actin cytoskeleton [24]. When the TJ barrier is disrupted, intestinal permeability is increased, and immune cells enter the gut mucosa through the paracellular pathway and release pro-inflammatory cytokines such as TNF-α, which induces inflammation. In addition, the weakness of TJs causes intestinal damage, such as ulceration, erosion, and apoptosis [11], consequently resulting in GI diseases. Decreased occludin and ZO-1 expression is observed in the intestinal tissue of PD patients, as well as in patients with GI disorders [6,25,26]. In our previous study, RJ alleviated colitis by protecting TJs in mice [11]. We confirmed that RJ treatment significantly inhibited the weakness of TJs in the colon of PD mice by regulating the expression of ZO-1 and occludin (Figure 7), which may be associated with the suppression of pro-inflammatory cytokines, especially TNF-α (Figure 8F). Therefore, RJ is thought to preserve the colon by inhibiting inflammation and protecting TJs.

LPS, a metabolite of Gram-negative bacteria, acts as an endotoxin and causes systemic inflammation [7]. LPS impairs the gut barrier by decreasing TJ proteins, such as occludin, and contributes to the production of pro-inflammatory cytokines [27]. Under LPS stimulation, enteric neurons and glial cells produce pro-inflammatory cytokines, such as IL-1β and TNF-α [7]. These pro-inflammatory cytokines and LPS can reach the brain via the vagus nerve and humoral pathways, such as lymphatic tissue and bloodstream [28,29]. Recent studies have shown that intraperitoneal or intravenous injection of LPS causes CNS inflammation [30], and TNF-α produced by LPS stimuli in the colon can move directly via the blood to the SN and lead to DA neuronal damage [27]. Moreover, LPS activates apoptosis and increases the aggregation and accumulation of α-synuclein in the colon [7], as well as the loss of DA neurons in the SN and motor impairment in mice [31]. Lewy bodies containing misfolded or aggregated α-synuclein in DA neurons are a major feature of PD [32]. In fact, α-synuclein aggregation is found in the brain and colon of PD patients [33]. The increased aggregation and propagation of α-synuclein induces the neurodegeneration of DA neurons in the SN, leading to PD motor dysfunction [34,35]. α-synuclein can be transported from the gut to the brain via the vagus nerve and the bloodstream, like LPS and cytokines [36]. In our study, the level of LPS was increased in the colon, serum, and SN of MPTP-treated mice (Figure 8A–C), and the levels of pro-inflammatory cytokines and α-synuclein were increased in both the colon and SN of MPTP-treated mice (Figure 8D–G and Figure 9). In addition, it is supposed that the increase in LPS may be involved in not only increasing the levels of pro-inflammatory cytokines and α-synuclein but also the loss of DA neurons (Figure 5), apoptosis (Figure 6), and motor dysfunction (Figure 4). However, these changes caused by MPTP were effectively alleviated by oral treatment with RJ, indicating that RJ prevents the production and conveyance of LPS, pro-inflammatory cytokines, and α-synuclein from the gut to the brain.

The BBB, a physiological barrier localized at the endothelium of cerebral blood vessels, maintains the homeostasis of the CNS by (i) inhibiting the inflow of toxic substrates to the brain from blood; (ii) mediating the transport of nutrients to the brain and the efflux of toxic metabolites from the brain; and (iii) controlling the migration of circulating immune cells [37]. The BBB is controlled by a neurovascular unit comprising pericytes embedded in the vascular basement membrane, perivascular microglial cells, astrocytes, and neurons [37]. Brain endothelial TJs (claudins, occludin, ZO proteins, etc.) play a major role in the BBB, such as the intestinal epithelial barrier, and claudin-3 and claudin-5 play an important role in the BBB, unlike the gut barrier [37]. AJs and TJs interact with each other in the brain endothelial cell barrier. VE-cadherin, a member of the AJs, regulates claudin-5 expression by inhibiting FoxO1 and β-catenin activity [37]. Previous studies have shown that MPTP injection disrupts the BBB, which induces the penetration of LPS, pro-inflammatory cytokines, and α-synuclein into the brain [34,38,39,40]. In the present study, MPTP increased the level of LPS in the colon and serum; however, oral administration of RJ significantly inhibited the increase in LPS in the SN tissue (*p* < 0.05, Figure 8). In addition, RJ inhibited the MPTP-induced reduction in VE-cadherin, occludin, and claudin-5 in the SN (*p* < 0.001, Figure 10), indicating that RJ prevented the increase in LPS in the brain by inhibiting MPTP-induced BBB collapse. Furthermore, the BBB-protective effect of RJ may be associated with the reduction in pro-inflammatory cytokines and α-synuclein levels in the SN of MPTP-treated mice (Figure 8 and Figure 9).

## 5. Conclusions

In the present study, RJ suppressed mitochondrial dysfunction and apoptosis in in vitro and in vivo PD models. Moreover, RJ protected against neuroinflammation and the accumulation of α-synuclein in the SN by preventing the collapse of the gut barrier and the migration of inflammatory factors and α-synuclein from the colon to the SN, thereby alleviated MPTP-induced motor dysfunction in mice. Therefore, RJ is expected to be potentially useful in the prevention and treatment of PD. However, further studies are required to determine the changes in microbiota composition (especially inflammation-related bacteria), functional compounds of RJ, detailed drug transport signaling, and potential in clinical practice to clarify the effects and mechanisms of the action of RJ.

## Figures and Tables

**Table 1:** Predicted anthraquinones of *Rumex japonicus* Houtt. from UPLC-Q-TOF-MS analysis.

RT (Min)	[M − H]^−^	[M − H]^−^ (*m*/*z*)	[M − H]^−^ (*m*/*z*)	Error (ppm)	Predicted Identity
	Molecular Formula	Measured	Calculated		
8.612	C_21_H_20_O_10_	431.0987	431.0984	−1.57	Emodin-8-glucoside
8.706	C_21_H_20_O_9_	415.1030	415.1035	0.35	Chrysophanol-8-O-β-d-glucoside (pulmatin)
8.756	C_15_H_8_O_6_	283.0255	283.0248	−1.49	Rhein
9.022	C_15_H_10_O_4_	253.0505	253.0506	0.36	Chrysophanol
9.049	C_22_H_22_O_10_	445.1140	445.1140	−0.94	Physcion-8-O-β-d-glucopyranoside
10.840	C_16_H_12_O_5_	283.0612	283.0612	1.15	Physcion
15.058	C_15_H_10_O_5_	269.0458	269.0455	−0.74	Aloe emodin
17.098	C_15_H_10_O_5_	269.0456	269.0455	−0.42	Emodin

## Data Availability

The data presented in this study are available in this manuscript.
